# What is the purpose of clinical trial monitoring?

**DOI:** 10.1186/s13063-022-06763-2

**Published:** 2022-10-01

**Authors:** Sharon B. Love, Victoria Yorke-Edwards, Elizabeth Ward, Rebecca Haydock, Katie Keen, Katie Biggs, Gosala Gopalakrishnan, Lucy Marsh, Lydia O’Sullivan, Lisa Fox, Estelle Payerne, Kerenza Hood, Garry Meakin

**Affiliations:** 1 MRC Clinical Trials Unit at UCL, 90 High Holborn, London, WC1V 6LJ UK; 2 Bristol Trials Centre (BTC), BRI Hub (CTEU Bristol), Level 7 Queens Building, Bristol Royal Infirmary, Marlborough Street, Bristol, BS2 8HW UK; 3 Nottingham Clinical Trials Unit, University of Nottingham, Building 42, University Park, Nottingham, NG7 2RD UK; 4 NHS Blood and Transplant Clinical Trials Unit, Long Road, Cambridge, CB2 0PT UK; 5 Clinical Trials Research Unit, ScHARR, The University of Sheffield, Regent Court, 30 Regent Street, Sheffield, S1 4DA UK; 6 Department of Surgery & Cancer, Imperial College London, Hammersmith Campus, 1st Floor, ICTEM Building, Du Cane Road, London, W12 0NN UK; 7 Centre for Trials Research, College of Biomedical & Life Sciences, Cardiff University, 6th Floor, Neuadd Meirionnydd, Heath Park, Cardiff, CF14 4YS UK; 8 Health Research Board - Trials Methodology Research Network, NUI Galway, University Road, Galway, Ireland; 9 ICR-CTSU, 15 Cotswold Road, Belmont, Sutton, Surrey, SM2 5NG UK; 10 Norwich Clinical Trials Unit, Norwich Medical School, University of East Anglia, Norwich, NR4 7TJ UK; 11 Centre for Trials Research, College of Biomedical & Life Sciences, Cardiff University, 7th Floor, Neuadd Meirionnydd, Heath Park, Cardiff, CF14 4YS UK

**Keywords:** Clinical trial monitoring, Trial conduct, Clinical trials methodology

## Abstract

**Background:**

The sources of information on clinical trial monitoring do not give information in an accessible language and do not give detailed guidance. In order to enable communication and to build clinical trial monitoring tools on a strong easily communicated foundation, we identified the need to define monitoring in accessible language.

**Methods:**

In a three-step process, the material from sources that describe clinical trial monitoring were synthesised into principles of monitoring. A poll regarding their applicability was run at a UK national academic clinical trials monitoring meeting.

**Results:**

The process derived 5 key principles of monitoring: keeping participants safe and respecting their rights, having data we can trust, making sure the trial is being run as it was meant to be, improving the way the trial is run and preventing problems before they happen.

**Conclusion:**

From the many sources mentioning monitoring of clinical trials, the purpose of monitoring can be summarised simply as 5 principles. These principles, given in accessible language, should form a firm basis for discussion of monitoring of clinical trials.

**Supplementary Information:**

The online version contains supplementary material available at 10.1186/s13063-022-06763-2.

## Background

The MRC-NIHR Trials Methodology Research Partnership (TMRP) Trial Conduct Working Group Data Quality and Monitoring (DQM) subgroup (hereafter “TMRP DQM”) comprises 13 people (authors of this paper) interested in improving the monitoring of clinical trials (four researchers, six trial managers, three other). Though location was not an exclusion when applications were requested for this subgroup, the current members are those involved in academic trials from the UK and Ireland, each involved in a variety of national and international trials. Despite each adapting our way of working from the guidance available, it was clear that we did not have a common monitoring strategy or understanding of terminology. We identified the need to go back to basics and define the purpose of monitoring. This will enable communication between researchers, regulators and trial teams and will give a strong easily explained foundation on which to build tools for clinical trial monitoring.

Clinical trial monitoring is an important aspect of clinical trial conduct. The International Council for Harmonisation of Technical Requirements for Pharmaceuticals for Human Use (ICH) Good Clinical Practice (GCP) E6(R2) [1] gives a definition of the purpose of clinical trial monitoring

*“5.18.1 Purpose*

*The purposes of trial monitoring are to verify that:*

*(a) The rights and well-being of human subjects are protected.*

*(b) The reported trial data are accurate, complete, and verifiable from source documents.*

*(c) The conduct of the trial is in compliance with the currently approved protocol/amendment(s), with GCP, and with the applicable regulatory requirement(s).”*

Though ICH GCP has used this underlying definition of the purpose of monitoring since 1996 [1], it is not written in accessible language and does not contain any explicit detailed guidance on monitoring activities [2]. Therefore, each clinical trial sponsor has taken this definition or the one in their own country’s legislation (for example [3]), interpreted it and created their own monitoring activities. This has led to wide variation in practices, as noted in several surveys of monitoring approaches [4–6].

Despite the recent shift towards a more risk-based approach, which has led to a re-focusing on monitoring, there remains little detailed guidance available on how it should be done. To facilitate the development of any guidance in the future, a clear understanding of the purpose of monitoring is essential.

This paper describes the process whereby the MRC-NIHR TMRP DQM extracted the purpose of monitoring, as described by pertinent sources, and from them defined the purpose of monitoring in accessible language. We hope that this paper can be used to formulate detailed guidance on how to monitor clinical trials.

## Methods

We chose the sources from guidance provided by organisations that any one of the TMRP DQM referred to when deciding how to run trials. Table 1 gives the organisations included and their aims. Each author reviewed an organisation’s publicly available online or downloadable information to extract any material on the purpose of monitoring. We did not contact the organisations. The documents reviewed included guidance documents, minutes of workshops, toolkits, blogs and reports of questions and answers (Additional file 1). In a second round, the information related to the purpose of monitoring was distilled into non-repetitive statements by pairs of authors. In the final round, all authors independently compared the purpose of monitoring across all sources and in the ensuing consensus meeting five key principles for the purpose of monitoring emerged.

**Table 1:** Descriptions of the organisations publishing on monitoring from the organisation website

Organisation and URL	Short name	Description
Clinical Trials Transformation Initiative https://www.ctti-clinicaltrials.org/	CTTI	Mission: To develop and drive adoption of practices that will increase the quality and efficiency of clinical trials. CTTI comprises more than 80 organizations from across the clinical trial enterprise. USA based private body, International
European Medicines Agency https://www.ema.europa.eu/en	EMA	The European Medicines Agency (EMA) is a decentralised agency of the European Union (EU) responsible for the scientific evaluation, supervision and safety monitoring of medicines in the EU. Public body, European Union
US Food and Drug Administration https://www.fda.gov/	FDA	The Food and Drug Administration is responsible for protecting the public health by ensuring the safety, efficacy, and security of human and veterinary drugs, biological products, and medical devices; and by ensuring the safety of our nation's food supply, cosmetics, and products that emit radiation. Public body, USA and further afield
Health Research Authority https://www.hra.nhs.uk/	HRA	[HRA] vision is for high-quality health and social care research that improves people’s health and wellbeing, and [HRA] core purpose is to protect and promote the interests of patients and the public in health and social care research. Public body, UK
International Council for Harmonisation of Technical Requirements for Pharmaceuticals for Human Use https://www.ich.org/	ICH	Brings together the regulatory authorities and pharmaceutical industry to discuss scientific and technical aspects of drug registration. Private body, International
Medicines and Healthcare products Regulatory Agency https://www.gov.uk/government/organisations/medicines–and–healthcare-products-regulatory-agency	MHRA	Regulates medicines, medical devices and blood components for transfusion in the UK. Public body, UK
National Institute for Health Research https://www.nihr.ac.uk/	NIHR	The UK's largest funder of health and care research and provide the people, facilities and technology that enables research to thrive. Working in partnership with the NHS, universities, local government, other research funders, patients and the public, [NIHR] deliver and enable world-class research that transforms people's lives, promotes economic growth and advances science. Public body, UK
TransCelerate Biopharma Inc https://www.transceleratebiopharmainc.com/	Trans-Celerate	Aim to collaborate across the global biopharmaceutical research and development community to identify, prioritize, design and facilitate implementation of solutions designed to drive the efficient, effective and high-quality delivery of new medicines. USA based, international
UK Trial Manager Network https://www.tmn.ac.uk/	UKTMN	Aims to facilitate the development of a well-trained, highly motivated, effective workforce of trial managers within the UK health care system who will make an important contribution to the efficient delivery of high quality clinical trials. Private body, UK

To obtain feedback on their value, we presented the five key principles covering the purpose of monitoring to delegates at the UKCRC Task and Finish Monitoring Group annual meeting 2021 (a national meeting of experts in conducting academic clinical trial monitoring in the UK) and asked “Are there any of these purposes of monitoring that you do not agree with?”, giving options to disagree with any principle or to answer “No, I agree with all 5 proposed principles”. In addition, delegates were asked to suggest any other potential “principles of monitoring” that we should consider (see Additional file 2).

We show the underlying text for these five principles and also give examples of which monitoring activities could happen within each of these principles. We also present the results of our poll of the annual meeting.

## Results

The results of our synthesis for the purpose of monitoring are given in Table 2 and include five principles. The purpose of monitoring is keeping participants safe and respecting their rights, having data we can trust, making sure the trial is being run as it was meant to be, improving the way the trial is run and preventing problems before they happen. In our poll at a national meeting of those monitoring clinical trials in the UK, 93 attendees voted, with 85% (80/93) agreeing with all 5 proposed principles for the purpose of monitoring (Table 3). One poll respondent suggested adding building relationships with sites as a principle.

**Table 2:** Purpose of monitoring key principles synthesised from major worldwide organisations

Purpose of monitoring	
Key principles in lay terms	Key principles in more technical language
Keeping participants safe and respecting their rights	To ensure, enhance and protect participants’ safety, wellbeing and rights.
Having data we can trust	Having the systems and processes (such as source data verification) to ensure that each data item is as reliable as is needed to be sure of the results of the trial
Making sure the trial is run as it was meant to be	Maintain trial integrity by ensuring the trial is conducted in compliance with the currently approved protocol/documentation, with GCP and with the applicable regulatory requirements
Improving the way the trial is run	Improving quality, conduct and efficiency in clinical trials.
Preventing problems before they happen	Contingency and mitigation planning for risks to both participant safety and trial processes.

**Table 3:** Results from poll about the principles of monitoring at national meeting of those monitoring academic clinical trials in the UK

Response to the question “Are there any of these that purposes of monitoring that you do not agree with?“ from 93 respondents	***N*** (%)
Keeping participants safe and respecting their rights	1 (1%)
Having data we can trust	2 (2%)
Making sure the trial was run as it was meant to be	3 (3%)
Improving the way the trial is run	8 (9%)
Preventing problems before they happen	4 (4%)
No – I agree with all 5 proposed principles	80 (86%)

Note that there were 93 respondents; some did not agree with more than one principle

Table 4 shows the clarification of our derivation of these principles by giving examples from the underlying sources. For example, the principle “having data we can trust” came in part from the CTTI statement “Ensuring that data quality is sufficient to answer study question”. In Additional file 3, we give the full text from all sources.

**Table 4:** Showing the link of the 5 principles of monitoring with the sources

Principle of monitoring in lay terms	Examples of principle source^**a**^	Source
Keeping participants safe and respecting their rights	Protecting the rights, safety, and welfare of subjects under the investigator’s care	FDA
	The risk associated with the IMP should also determine the trial procedures for monitoring the safety of participants.	MHRA
	Inspectors should verify procedures for reviewing and communicating findings that could adversely affect the safety of subjects.	EMA
	[…] safety must be monitored in all trials and therefore the need for formal procedures to cover early stopping for safety reasons should always be considered.	ICH E9 3.4
	[…] adequate oversight and monitoring during the trial will help ensure that trial subject safety is maintained throughout the trial.	NIHR
Having data we can trust	Careful attention to quality during trial planning, investigator training, trial monitoring and audit will help consistently achieve trial quality required.	ICH
	Ensuring that data quality is sufficient to answer study question.	CTTI
	Monitoring strategies, tailored to risks, should permit timely oversight and be focused on critical processes and critical data.	TransCelerate
	Appropriate planning before the trial and adequate oversight and monitoring during the trial will help ensure that trial subject safety is maintained throughout the trial and that there is accurate reporting of results at its conclusion.	NIHR
	Ensure … data quality across sites.	FDA
Making sure the trial was run as it was meant to be	[…] preventing or mitigating important and likely sources of error in the conduct, collection, and reporting of critical data and processes necessary for human subject protection and trial integrity.	FDA
	[…] perform checks that include: verification that trial documents exist, assessment of the site’s understanding of, and compliance with the protocol and trial procedures […]	NIHR
	Essential documents are those ‘documents which individually and collectively permit evaluation of the conduct of a trial and the quality of the data produced’ and they serve to demonstrate compliance with the principles of GCP and regulatory requirements.	UKTMN
	Investigators are appropriately selected, trained and supported to complete the proposed clinical trial (MHRA).	MHRA
Improving the way the trial is run	Monitoring during the trial will help ensure that trial subject safety is maintained throughout the trial and that there is accurate reporting of results at its conclusion.	NIHR
	Monitoring strategies, tailored to risks, should permit timely oversight and be focused on Critical Processes and Critical Data. Notably, Investigators are responsible for their site’s data quality and are expected to partner with the Sponsor to address, resolve, and prevent issues.	TransCelerate
	Chief investigators are responsible for the overall conduct of a research project including adhering to the agreed procedures and arrangements for reporting (e.g. progress reports, safety reports) and for monitoring the research, including its conduct, the participants’ safety and well-being and the ongoing suitability of the approved proposal or protocol in light of adverse events or other developments.	HRA
	Moreover, a risk-based approach is dynamic, more readily facilitating continual improvement in trial conduct and oversight. For example, monitoring findings should be evaluated to determine whether additional actions (e.g. training of clinical investigator and site staff, clarification of protocol requirements) are necessary to ensure human subject protection and data quality across sites.	FDA
	Maximizing efficiency for minimal resource use	CTTI
Preventing problems before they happen	Sponsors should prospectively identify critical data and processes, then perform a risk assessment to identify and understand the risks that could affect the collection of critical data or the performance of critical processes, and then develop a monitoring plan that focuses on the important and likely risks to critical data and processes.	FDA
	A Trial Monitoring Plan will be developed and agreed by the Trial Management Group (TMG), TSC and CI based on the trial risk assessment which may include on site monitoring. This will be dependent on a documented risk assessment of the trial.	HRA
	The sponsor should develop a monitoring plan that is tailored to the specific human subject protection and data integrity risks of the trial. The plan should describe the monitoring strategy, the monitoring responsibilities of all the parties involved, the various monitoring methods to be used, and the rationale for their use. The plan should also emphasize the monitoring of critical data and processes.	ICH
	Once developed, the risk assessment and associated management/monitoring plans would form the basis of a common understanding by all stakeholders on the risks for that trial and facilitate a risk-proportionate approach to the trial activities.	MHRA
	Risk-based monitoring: An adaptive approach [to clinical trial monitoring] that directs monitoring focus and activities to the evolving areas of greatest need which have the most potential to impact subject safety and data quality.	TransCelerate

^a^This is not exhaustive. It is a selection of examples from a selection of sources for illustration

We have given examples in Table 5 from sources and from our experience to show how each principle may be mitigated by monitoring. For example, if the principle of monitoring is to keep participants safe and respect their rights, then we should monitor to ensure the consent is valid.

**Table 5:** Examples of monitoring for each principle

Principle	Examples
Keeping participants safe and respecting their rights	Ensure valid consent by checking the consent forms are completed correctly Ensure data available by checking that the expected data have been entered onto the database Look regularly at the amassed safety data and protocol compliance. Present to regulatory authorities, CI, DMC, TMG, REC and safety review committee and act upon their direction/advice. This may result in a change to the protocol or trial conduct.
Having data we can trust	Develop a risk-based monitoring plan that identifies critical data and processes and focusses on ensuring their accuracy and integrity. Perform on-site and/or remote monitoring and/or central monitoring, where required. Build quality into the scientific and operational design and conduct of clinical trials including an audit programme to evaluate processes relating to data quality and perform root cause analysis and establish corrective and preventative actions where significant deficiencies are detected.
Making sure the trial was run as it was meant to	Ensure site staff are appropriately trained and qualified to deliver their role on the trial and to follow trial specific procedures and processes (e.g. monitoring delegation and training logs, checking CVs). Collect and check protocol deviations/non-conformances and ensure systems are in place to mitigate risks of these happening again such as retraining and providing working practice documents. Ensure a monitoring plan is in place that allows timely evaluation of significant issues identified.
Improving the way the trial is run	Ensure the trial adheres to GCP, protocol and ethical/regulatory guidelines. Ensure quality measure are reached by providing adequate staffing and resources. Continuously monitor and respond to issues in a timely manner with corrective actions when required/appropriate
Preventing problems before they happen	Base the monitoring plan and activities on a risk assessment Undertake a risk assessment to assess the potential risks and puts things in place to prevent and monitor these risks.

## Discussion

We have clarified the purpose of monitoring in accessible language from pertinent sources. This will enable communication between those carrying out clinical trial monitoring and facilitate the building of clinical trial monitoring tools on a strong easily communicated foundation.

Clinical trial monitoring is a crucial part of trial conduct, improving the safety of the participants, the quality of the data and the trial integrity. Clinical trial monitoring is conducted by monitors, quality assurance teams and by trial managers [5]. Guidance on trial monitoring is spread amongst different sources within and between organisations, is often in technical language and does not describe practicalities of how to achieve the monitoring aims. The differing specialities conducting monitoring and the disparity and high-level content of the sources mean there is scope for misunderstanding and communication errors. This could easily lead to poor quality monitoring which could jeopardise the protection of the rights and safety of patients and the data and trial integrity. Also, misunderstanding could lead to over-monitoring for no additional value, placing additional burden on research teams and sites and increasing the cost of undertaking research. It is difficult to have methodology research discussions, and therefore to deliver monitoring tools, without agreement of a common basis which multiple disciplines can all understand. It is difficult for grant committees to understand the value of monitoring research when there is no easily understandable consensus on why we need to do good clinical trial monitoring. The differing language and range of ideas on clinical trial monitoring are confusing and unhelpful. The variability in language and practices this range has caused, as evidenced by 13 people in the UK who were experienced in clinical trial monitoring finding it difficult to communicate and find common ground, is limiting progress in clinical trial monitoring. We have synthesised the sources to describe the purpose of monitoring in five key principles described in both technical and lay language (Table 2). We intend this to be a basis for making monitoring more accessible.

The purpose of monitoring principles presented in this paper are active, continual and responsive to monitoring findings. They are focussed on the conduct of a real trial rather than based on theory. They are aimed at monitoring to intervene and make trials better rather than certifying what has already been done. Monitoring is an integral part of trial conduct rather than an add-on. Our first three principles link with parts a, b and c of the ICH definition of monitoring (see methods) but the idea of improving trials or preventing problems before they happen is not part of the ICH definition. These are important aspects of monitoring, as finding an issue at one site early in the trial means that training can be given to the other sites and the overall trial is improved to the benefit of current and future patients. Stating improvement and prevention in our principles will help those running trials to address improvement to the trial and prevent poor conduct.

The FDA provided particularly valuable sources for this paper as they have strongly advocated risk-based monitoring and have had to be clear in their documents as they are extensively used worldwide.

Supplementary Table 2 lists the sources used. Although the thirteen authors all have experience of clinical trial monitoring in the UK and Irish academic setting, relevant and valuable sources of information may have been missed. The first selection of useful text from the individual documents within each source was done by one author and the second phase by groups of two or three. Only the final phase was completed by all authors together. These are limitations in the study but it also exemplifies how those devising monitoring policies for their trials and those carrying out monitoring activities must refer to multiple sources. The 86% vote of agreement with the five principles at the UK monitoring meeting corroborates the work done.

Though there is a limitation due to this work using world-wide English language sources, being led by UK and Ireland researchers and being corroborated in a UK national meeting, it is based on sources that are used by many. In future, it would be good to extend this to more of the clinical trial monitoring community world-wide.

Although building relationships with sites has been noted as a useful part of monitoring in publications [7–9], this did not come through from the sources reviewed and was only mentioned by one poll respondent at the national monitoring meeting. At present, we do not note it as one of the main five principles but further work may add principles.

As each group leading clinical trials has created their own monitoring strategy and language, there have been difficulties in communicating between groups [5]. Our principles clarifying the purpose of monitoring should improve communication and enable the monitoring community to start producing clear monitoring practical guidelines and to start sharing tools. These principles should be considered when undertaking risk assessments, developing monitoring plans and carrying out monitoring activities.

## Conclusions

From the many sources mentioning monitoring of clinical trials, the purpose of monitoring can be summarised simply as (i) keeping participants safe and respecting their rights, (ii) having data we can trust, (iii) making sure the trial is being run as it was meant to be, (iv) improving the way the trial is run and (v) preventing problems before they happen.

## Supplementary Information

**Additional file 1.** Sources used for the paper.

**Additional file 2.** Questions used in UKCRC Task and Finish Monitoring Group annual meeting 9 June 2021.

**Additional file 3.** File giving a complete version of Table S3.

## Data Availability

All data and material are given in paper and supplementary files.

## Abbreviations

COVID-19: Coronavirus disease 2019
CTTI: Clinical Trials Transformation Initiative
CTU: Clinical Trials Unit
EMA: European Medicines Agency
FDA: US Food and Drug Administration
GCP: Good Clinical Practice
HRA: UK Health Research Authority
ICH: International Council for Harmonisation of Technical Requirements for Pharmaceuticals for Human Use
MHRA: Medicines and Healthcare products Regulatory Agency
NIHR: National Institute for Health Research
UKTMN: UK Trial Managers Network
