# Supplementary material for: What is the purpose of clinical trial monitoring?
Source: Trials. 2022 Oct 1;23:836. doi: 10.1186/s13063-022-06763-2 (PMC9526458; doi:10.1186/s13063-022-06763-2)
Supplement: Supplementary file 1 — Additional file 1. Sources used for the paper. [file 13063_2022_6763_MOESM1_ESM.docx]

Additional File 1

Sources used

| **Source** | **Reference** |
| --- | --- |
| Clinical Trials Transformation Initiative | Quality Objectives of Monitoring Workstream 2 Final Report  (<https://www.ctti-clinicaltrials.org/files/monitoringws2finalreport.pdf>) |
| European Medicines Agency | Guidance on the management of clinical trials during the COVID-19 (coronavirus) pandemic 2020  https://ec.europa.eu/health/  sites/health/files/files/eudralex/vol-10/guidanceclinicaltrials_covid19_en.pdf  Accessed 10_Jun_2020.  Annex IV – to guidance for the conduct of good clinical practice inspections – sponsor and CRO  <https://ec.europa.eu/health/sites/default/files/files/eudralex/vol-10/eudralex_vol10_chapter4_guidance-conduct_annex4.pdf>  Accessed 31Aug2021 |
| US Food and Drug Administration | Guidance for Industry Oversight of Clinical Investigations — A Risk-Based Approach to Monitoring (August 2013)  <https://www.fda.gov/media/77765/download>  Accessed 31Aug2021  A Risk-Based Approach to Monitoring of Clinical Investigators Questions and Answers Guidance for Industry (Draft Guidance March 2019)  Establishment and Operation of Clinical Trial Data Monitoring Committees (March 2006)  FDA Guidance on Conduct of Clinical Trials of Medical Products during COVID-19 Public Health Emergency (March 2020) |
| Health Research Authority | Making changes to a research study to manage the impact of COVID-19  https://www.hra.nhs.uk/covid-19-research/covid-19-  guidance-sponsors-sites-and-researchers/#monitoring  Accessed 18_Jun_2020.  UK Policy Framework for Health and Social Care Research  [UK Policy Framework for Health and Social Care Research - Health Research Authority (hra.nhs.uk)](https://www.hra.nhs.uk/planning-and-improving-research/policies-standards-legislation/uk-policy-framework-health-social-care-research/uk-policy-framework-health-and-social-care-research/)  Accessed 30Jun2020  HRA Protocol guidance and template for use in a Clinical Trial of an Investigation Medicinal Product (CTIMP)  [Protocol - Health Research Authority (hra.nhs.uk)](https://www.hra.nhs.uk/planning-and-improving-research/research-planning/protocol/)  Access 30Jun2020 |
| International Council for Harmonisation of Technical Requirements for Pharmaceuticals for Human Use | ICH harmonised guideline integrated addendum to ICH E6(R1): Guideline for Good Clinical Practice ICH E6(R2) ICH Consensus Guideline <https://ichgcp.net> (E2, E3, E5, E9, E10, E17) |
| Medicines and Healthcare products Regulatory Agency | Managing clinical trials during coronavirus (COVID-19) 2020  https://www.gov.uk/guidance/managing-clinical-trials-during-coronaviruscovid-19  Accessed 18_Jun_2020.  Risk-adapted Approaches to the Management of Clinical Trials of Investigational Medicinal Products  <https://assets.publishing.service.gov.uk/government/uploads/system/uploads/attachment_data/file/343677/Risk-adapted_approaches_to_the_management_of_clinical_trials_of_investigational_medicinal_products.pdf>  Accessed 31Aug2021  <https://forums.mhra.gov.uk/showthread.php?1675-MHRA-produced-FAQs-for-monitoring>  Accessed 22Dec2021  MHRA Good clinical practice guide 2012  <https://mhrainspectorate.blog.gov.uk/2017/11/16/risk-adaption-in-clinical-trials-of-investigational-medicinal-products-ctimps/>  Accessed 22Dec2021  <https://assets.publishing.service.gov.uk/government/uploads/system/uploads/attachment_data/file/343677/Risk-adapted_approaches_to_the_management_of_clinical_trials_of_investigational_medicinal_products.pdf>  Accessed 22Dec2021 |
| National Institute for Health Research | Clinical trials toolkit – routemap – Trial Management & Monitoring  <https://www.ct-toolkit.ac.uk/routemap/trial-management-and-monitoring/>  Accessed 31Aug2021 |
| TransCelerate Biopharma Inc | Risk based monitoring interactive guide  <https://www.transceleratebiopharmainc.com/rbminteractiveguide/what-is-risk-based-monitoring-rbm/introduction/>  Accessed 31Aug2021 |
|  | Risk based monitoring position paper  <http://www.transceleratebiopharmainc.com/wp-content/uploads/2016/01/TransCelerate-RBM-Position-Paper-FINAL-30MAY2013.pdf.pdf>  Accessed 31Aug2021 |
|  | Measuring the impact of risk based monitoring  <http://transceleratebiopharmainc.com/wp-content/uploads/2019/12/RBM-Metrics-Report_December-2019.pdf>  Accessed 31Aug2021 |
|  | Risk-Based-Quality-Management QTL 2017  <http://www.transceleratebiopharmainc.com/wp-content/uploads/2017/09/Risk-Based-Quality-Managment.pdf>  Accessed 31Aug2021 |
| UK Trial Manager Network | The Guide to Efficient Trial Management : Effectively managing clinical trials, Sixth Edition (2018)  <https://cdn.ymaws.com/www.tmn.ac.uk/resource/resmgr/tmn_guide/uktmng2.web.pdf>  Accessed 30Jun2020 |
