# Supplementary material for: What is the purpose of clinical trial monitoring?
Source: Trials. 2022 Oct 1;23:836. doi: 10.1186/s13063-022-06763-2 (PMC9526458; doi:10.1186/s13063-022-06763-2)
Supplement: Supplementary file 2 — Additional file 2. Questions used in UKCRC Task and Finish Monitoring Group annual meeting 9 June 2021. [file 13063_2022_6763_MOESM2_ESM.docx]

**Questions used in UKCRC Task and Finish Monitoring Group annual meeting 9June 2021**

Question: Are there any of these purposes of monitoring that you do not agree with?

Results:


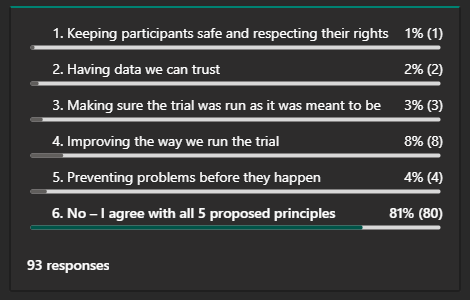


(note percentages given automatically in software above are as if those answering could only make one answer whereas they could make multiple answers. The percentages given in the paper are correct)

Question: Are there any other principles of monitoring that we should include?

Answer: building relationships with sites (one answer received)
