# Supplementary material for: What is the purpose of clinical trial monitoring?
Source: Trials. 2022 Oct 1;23:836. doi: 10.1186/s13063-022-06763-2 (PMC9526458; doi:10.1186/s13063-022-06763-2)
Supplement: Supplementary file 3 — Additional file 3. File giving a complete version of Table S3. [file 13063_2022_6763_MOESM3_ESM.docx]

Additional File 3

**Supplementary version of Table 3** Purpose in lay terms and the underlying information from international sources

Note: Multiple source reference are in one row if they use exactly the same words or different words that seem to mean the same thing. If the words are different, the different words are also written into the principles column.

| **Purpose of monitoring in lay terms** | **Principles** | **international source reference** | | | | | | | | |
| --- | --- | --- | --- | --- | --- | --- | --- | --- | --- | --- |
|  |  | **CTTI** | **EMA** | **FDA** | **HRA** | **ICH** | **MHRA** | **NIHR** | **Trans-Celerate** | **UKTMN** |
| Keeping participants safe and respecting their rights | **Protocol compliance** |  |  |  |  |  |  |  |  |  |
|  | Protocol compliance needs to be monitored | * |  |  |  | * | * | * | * |  |
|  | Safety |  |  |  |  |  |  |  |  |  |
|  | Subjects treated ethically  Ensure adequate protection of the rights, welfare, and safety of human subjects | * |  | * |  | * |  |  |  |  |
|  | Inspectors may verify procedures for safety reporting  DSUR assures regulators that sponsor is adequately monitoring and evaluating the safety profile  Adverse event report to regulators |  | * |  |  | * |  |  |  |  |
|  | Urgent safety measures may be made to protect participants |  |  |  |  |  | * |  |  |  |
|  | Ensuring participant safety during the trial is the more important that the interests of science and society |  |  |  | * |  | * | * |  |  |
|  | Monitoring should enhance the safety of participants |  |  |  |  |  |  |  | * |  |
|  | communication |  |  |  |  |  |  |  |  |  |
|  | Communication  Procedures for reviewing and communicating safety reports | * | * |  | * |  | * |  |  |  |
|  | Inspectors may verify procedures for ……. communication |  | * |  |  |  |  |  |  |  |
|  | Risk or/and benefit |  |  |  |  |  |  |  |  |  |
|  | Means by which trial can be terminated if it presents too much risk | * |  |  |  | * | * |  |  |  |
|  | Close monitoring to quickly find issues  Safety and efficacy need to be monitored and (if necessary) should result in actions to modify the protocol to reduce risk |  | * |  |  | * |  |  |  |  |
|  | Safety risks to participants should be included in trial risk assessments and safety monitoring plans |  |  |  |  |  | * |  |  |  |
|  | Sponsors should protect participants by prospectively identifying important likely sources of risk to participant safety and any processes necessary to protect participants and focus oversight on these |  |  | * |  |  |  |  |  |  |
|  | Keeping safe throughout the trial |  |  |  |  |  |  |  |  |  |
|  | Maintaining safety throughout the trial |  |  |  |  |  |  | * |  |  |
|  | Evaluation of whether a protocol is safe should continue throughout trial |  |  |  | * |  |  |  |  |  |
| Having data we can trust | Consider quality throughout the life of a trial |  |  |  |  |  |  |  |  |  |
|  | Build quality into the scientific and operational design and conduct of clinical trials. | * |  |  |  |  |  |  |  |  |
|  | Careful attention to quality during trial planning, investigator training, trial monitoring and audit will help consistently achieve trial quality required for a successful MRCT |  |  |  |  | * |  |  |  |  |
|  | Appropriate planning before the trial and adequate oversight and monitoring during the trial will help ensure that trial subject safety is maintained throughout the trial and that there is accurate reporting of results at its conclusion. |  |  |  |  |  |  | * |  |  |
|  | Operational checks (e.g., on-site, remote, and centralized monitoring) and statistical surveillance can identify important data quality issues at a point at which corrective action is feasible | * |  |  |  |  |  |  |  |  |
|  | Monitoring involves overseeing the progress of the trial. |  |  |  |  |  |  |  |  | * |
|  | Sponsors should use the results of the risk assessment in developing the monitoring plan (e.g., determining which risks may be addressed through monitoring, determining the types and intensity of monitoring activities best suited to addressing these risks). A monitoring plan ordinarily should focus on preventing or mitigating important and likely risks, identified by the risk assessment, to critical data and processes. |  |  | * |  |  |  |  |  |  |
|  | Identify key data that impacts results |  |  |  |  |  |  |  |  |  |
|  | The sponsor should develop a monitoring plan that is tailored to the specific human subject protection and data integrity risks of the trial |  |  |  |  | * |  |  |  |  |
|  | For each clinical trial, the sponsor should develop a monitoring plan that describes the monitoring methods, responsibilities, and requirements for the trial. The monitoring plan should include a brief description of the study, its objectives, and the critical data and study procedures, with particular attention to data and procedures that are unusual in relation to clinical routine and require training of study site staff. |  |  | * |  |  |  |  |  |  |
|  | Focus on what matters. | * |  |  |  |  |  |  |  |  |
|  | Sponsors should prospectively identify critical data and processes, then perform a risk assessment to identify and understand the risks that could affect the collection of critical data or the performance of critical processes, and then develop a monitoring plan that focuses on the important and likely risks to critical data and processes. |  |  | * |  |  |  |  |  |  |
|  | Review of primary outcome data. |  |  |  |  |  |  |  |  | * |
|  | Emphasis should be placed on the quality of data required to meet the trial objectives and to obtain reliable results. | * |  |  |  |  |  |  |  |  |
|  | The major quality objectives for monitoring of clinical trials are related to ensuring integrity of data generated in clinical trials, and thus the reliability of the study findings. Monitoring objectives proposed by meeting attendees…..ensuring data quality is sufficient to answer the study question. Ongoing data monitoring provides assurance that trial participants’ safety will be protected (e.g., a trial will be terminated if it presents an unreasonable and significant risk) and that the data gathered during a trial will be fit for purpose. |  |  |  |  |  |  |  | * |  |
|  | Monitoring strategies, tailored to risks, should permit timely oversight and be focused on Critical Processes and Critical Data.  It is recommended that the monitoring plan specifies the level of SDV required……..This will vary from trial to trial based on the risk assessment that would identify the data that are critical to the reliability of the results and subject safety. |  |  |  |  |  | * |  |  |  |
|  | Evaluate for systematic or significant errors in data collection and reporting at a site or across sites; or potential data manipulation or data integrity problems. |  |  |  |  | * |  |  |  |  |
|  | The purposes of trial monitoring are to verify that…. the reported trial data are accurate, complete, and verifiable from source documents. | * |  |  |  |  |  |  |  |  |
|  | Quality is defined as the absence of errors that matter (i.e. errors that have a meaningful impact on patient safety or interpretation of results) | * |  |  |  |  |  |  |  |  |
|  | Oversee processes and monitor key data to ensure quality |  |  |  |  |  |  |  |  |  |
|  | Monitoring strategies, tailored to risks, should permit timely oversight and be focused on critical processes and critical data |  |  |  |  |  |  |  | * |  |
|  | A risk-based approach to monitoring does not suggest any less vigilance in oversight of clinical investigations. Rather, it focuses sponsor oversight activities on preventing or mitigating important and likely risks to data quality and to processes critical to human subject protection and trial integrity. The overarching goal of this guidance is to enhance human subject protection and the quality of clinical trial data by focusing sponsor oversight on the most important aspects of study conduct and reporting. Sponsors of clinical investigations involving human drugs, biological products, medical devices, and combinations thereof are required to provide oversight to ensure adequate protection of the rights, welfare, and safety of human subjects and the quality of the clinical trial data submitted to FDA. |  |  | * |  |  |  |  |  |  |
|  | The sponsor’s oversight and monitoring can be regarded to encompass all the activities undertaken by the sponsor during the conduct of the trial that are there to ensure the subjects rights and well being are protected, the reliability of the trial data and hence the trial results and that the trial is conducted in accordance with the legislation. |  |  |  |  |  | * |  |  |  |
|  | Appropriate planning before the trial and adequate oversight and monitoring during the trial will help ensure that trial subject safety is maintained throughout the trial and that there is accurate reporting of results at its conclusion. |  |  |  |  |  |  | * |  |  |
|  | Centralized monitoring processes provide additional monitoring capabilities that can complement and reduce the extent and/or frequency of on-site monitoring and help distinguish between reliable data and potentially unreliable data.  Review, that may include statistical analyses, of accumulating data from centralized monitoring can be used to:…..examine data trends such as the range, consistency, and variability of data within and across sites. And evaluate for systematic or significant errors in data collection and reporting at a site or across sites. If noncompliance that significantly affects or has the potential to significantly affect human subject protection or reliability of trial results is discovered, the sponsor should perform a root cause analysis and implement appropriate corrective and preventive actions. |  |  |  |  | * |  |  |  |  |
|  | Establish an audit system, as part of a quality assurance system, in order to evaluate activities related to clinical trials. |  | * |  |  |  |  |  |  |  |
|  | Accurately represent key data |  |  |  |  |  |  |  |  |  |
|  | Accurate representation of key date, as manifested by proper reporting of data, reliability of results, ability of data to address the study question, reproducibility of study results. | * |  |  |  |  |  |  |  |  |
|  | The purposes of trial monitoring are to verify that….the reported trial data are accurate, complete, and verifiable from source documents.….ensuring high quality of study design and conduct is of paramount importance to ensure the study results are interpretable. |  |  |  |  | * |  |  |  |  |
|  | Appropriate planning before the trial and adequate oversight and monitoring during the trial will help ensure that trial subject safety is maintained throughout the trial and that there is accurate reporting of results at its conclusion. |  |  |  |  |  |  | * |  |  |
|  | In summary, the purpose of monitoring clinical trials is to verify that:  …… the reported trial data are accurate, complete and verifiable against the source documents. |  |  |  |  |  | * |  |  |  |
|  |  |  |  |  |  |  |  |  |  |  |
| Making sure the trial was run as it was meant to be | Trial integrity |  |  |  |  |  |  |  |  |  |
|  | preventing or mitigating important and likely sources of error in the conduct, collection, and reporting of critical data and processes necessary for human subject protection and trial integrity. |  |  | * |  |  |  |  |  |  |
|  | “The monitor(s) in accordance with the sponsor’s requirements should ensure that the trial is conducted and documented properly” |  |  |  |  | * |  |  |  |  |
|  | Protocol compliance |  |  |  |  |  |  |  |  |  |
|  | Monitoring is a quality control tool for determining whether study activities are being carried out as planned (FDA)  The participants were in accord with the findings concerning the major quality objectives—patient safety, accuracy of key data, and **compliance....**  (CTTI) | * |  | * |  |  |  |  |  |  |
|  | To verify that processes are consistently followed and activities are consistently documented to ensure high-quality trial conduct and protocol compliance |  |  |  |  |  | * |  |  |  |
|  | […] perform checks that include: verification that trial documents exist, assessment of the site’s understanding of, and compliance with the protocol and trial procedures,[…] |  |  |  |  |  |  | * |  |  |
|  | The conduct of the trial is in compliance with the currently approved protocol/amendment(s)… (MHRA and ICH)  to evaluate trial conduct and compliance with the protocol, SOPs, GCP, and the applicable regulatory requirements (ICH)  [requires a sponsor to ensure] that the investigation(s) is conducted in accordance with the general investigational plan and protocols contained in the IND. (FDA) |  |  | * |  | * | * |  |  |  |
|  | Review, that may include statistical analyses, of accumulating data from centralized monitoring can be used to: a) identify […] protocol deviations |  |  |  |  | * |  |  |  |  |
|  | Regulatory compliance |  |  |  |  |  |  |  |  |  |
|  | Essential documents are those ‘documents which individually and collectively permit evaluation of the conduct of a trial and the quality of the data produced’ and they serve to demonstrate compliance with the principles of GCP and regulatory requirements |  |  |  |  |  |  |  |  | * |
|  | The conduct of the trial is in compliance with […] GCP and with the applicable regulatory requirements. |  |  |  |  | * | * |  |  |  |
|  | Critical process and procedures |  |  |  |  |  |  |  |  |  |
|  | … assess adherence to the protocol, ensure the ongoing implementation of appropriate data entry and quality control procedures, and in general assess adherence to good clinical practices (FDA)  ...ensure adherence to standard operating procedures, and training in processes and procedures (ICH) |  |  | * |  | * |  |  |  |  |
|  | Quality improvement, using the monitoring process not only to identify errors but, if possible, to also provide a means by which the errors may be corrected via site training, protocol amendment, and so forth (EMA)  A risk-based monitoring strategy addresses the question: What are the critical processes and critical data for this trial and how best can any risks and/or vulnerabilities identified in these areas be managed or mitigated in order to avoid errors that matter? (NIHR)  The [monitoring] plan should also emphasize the monitoring of critical data and processes. (ICH) | * |  |  |  | * |  | * |  |  |
|  | Staff and training |  |  |  |  |  |  |  |  |  |
|  | Investigators are appropriately selected, trained and supported to complete the proposed clinical trial (MHRA)  Ensure that training, resources and facilities remain adequate (UKTMN)  Verifying that the investigator has adequate qualifications and resources (see 4.1, 4.2, 5.6) and remain adequate throughout the trial period, that facilities, including laboratories, equipment, and staff, are adequate to safely and properly conduct the trial and remain adequate throughout the trial period. (ICH) |  |  |  |  | * | * |  |  | * |
|  | …provide a means by which the errors may be corrected via site training (CTTI) | * |  |  |  |  |  |  |  |  |
|  | [Monitoring personnel] Must be familiar with the IMPs, the protocol, written informed consent form & GCP and regulatory requirements (MHRA)  Monitoring should be performed by someone with appropriate scientific/clinical knowledge who is familiar with the IMP, protocol, documents given to participants, GCP and applicable SOPs and regulatory requirements (UKTMN) |  |  |  |  |  | * |  |  | * |
| Improving the way we run the trial | Risk Based Monitoring Initiative could lead to improvement in data quality and patient safety for clinical trials and reduction in effort expended on low-value activities.  In addition, adoption of the Initiative’s approach to Risk Based Monitoring could lead to cost reductions through more focused centralized monitoring activity and targeted on-site monitoring. (TransCelerate)  Monitoring during the trial will help ensure that trial subject safety is maintained throughout the trial and that there is accurate reporting of results at its conclusion. (NIHR)  The sponsor should determine the appropriate extent and nature of monitoring. The determination of the extent and nature of monitoring should be based on considerations such as the objective, purpose, design, complexity, blinding, size, and endpoints of the trial. In general there is a need for on-site monitoring, before, during, and after the trial; however in exceptional circumstances the sponsor may determine that central monitoring in conjunction with procedures such as investigators’ training and meetings, and extensive written guidance can assure appropriate conduct of the trial in accordance with GCP. (ICH E6 5.18.3)  “Chief investigators are responsible for the overall conduct of a research project”  “adhering to the agreed procedures and arrangements for reporting (e.g. progress reports, safety reports) and for monitoring the research, including its conduct, the participants’ safety and well-being and the ongoing suitability of the approved proposal or protocol in light of adverse events or other developments”  “Research teams are responsible for demonstrating to chief investigators and sponsors their suitability to conduct the research” (HRA)  Moreover, a risk-based approach is dynamic, more readily facilitating continual improvement in trial conduct and oversight. For example, monitoring findings should be evaluated to determine whether additional actions (e.g., training of clinical investigator and site staff, clarification of protocol requirements) are necessary to ensure human subject protection and data quality across sites.  monitoring refers to the methods used by sponsors of investigational studies, or CROs delegated responsibilities for the conduct of IND studies, to oversee the conduct of, and reporting of data from, clinical investigations, including appropriate CI supervision of study site staff and third party contractors. Monitoring activities include communication with the CI and study site staff; review of the study site’s processes, procedures, and records; and verification of the accuracy of data submitted to the sponsor. (FDA)  Flexibility Maximizing efficiency for minimal resource use (CTTI) | * |  | * | * | * |  | * | * |  |
| Preventing problems before they happen | Risk assessment driven monitoring plan |  |  | * | * | * | * |  | * |  |
|  | Review site processes and procedures to ensure that the site continues to be trained/qualified to deliver comparable conditions in clinical operations. | * |  |  |  |  |  |  |  |  |
